# Detection of solid and subsolid pulmonary nodules with lung MRI: performance of UTE, T1 gradient-echo, and single-shot T2 fast spin echo

**DOI:** 10.1186/s40644-023-00531-4

**Published:** 2023-02-16

**Authors:** Felipe Sanchez, Pascal N. Tyrrell, Patrick Cheung, Chinthaka Heyn, Simon Graham, Ian Poon, Yee Ung, Alexander Louie, May Tsao, Anastasia Oikonomou

**Affiliations:** 1 Department of Medical Imaging, Sunnybrook Health Sciences Centre, University of Toronto, 2075 Bayview Avenue, Toronto, Ontario M4N 3M5 Canada; 2 Department of Medical Imaging, Department of Statistical Sciences, Institute of Medical Science, University of Toronto, 263 McCaul Street, Toronto, Ontario M5T 1WT Canada; 3 Department of Radiation Oncology, Sunnybrook Health Sciences Centre, University of Toronto, 2075 Bayview Avenue, Toronto, Ontario M4N 3M5 Canada; 4 Physical Sciences Platform of Sunnybrook Research Institute, Department of Medical Biophysics, University of Toronto, 2075 Bayview Avenue, Toronto, Ontario M4N 3M5 Canada

**Keywords:** Lung MRI, Pulmonary nodule, UTE, VIBE, HASTE

## Abstract

**Background:**

Although MRI is a radiation-free imaging modality, it has historically been limited in lung imaging due to inherent technical restrictions. The aim of this study is to explore the performance of lung MRI in detecting solid and subsolid pulmonary nodules using T1 gradient-echo (GRE) (VIBE, Volumetric interpolated breath-hold examination), ultrashort time echo (UTE) and T2 Fast Spin Echo (HASTE, Half fourier Single-shot Turbo spin-Echo).

**Methods:**

Patients underwent a lung MRI in a 3 T scanner as part of a prospective research project. A baseline Chest CT was obtained as part of their standard of care. Nodules were identified and measured on the baseline CT and categorized according to their density (solid and subsolid) and size (> 4 mm/ ≤ 4 mm). Nodules seen on the baseline CT were classified as present or absent on the different MRI sequences by two thoracic radiologists independently. Interobserver agreement was determined using the simple Kappa coefficient. Paired differences were compared using nonparametric Mann-Whitney U tests. The McNemar test was used to evaluate paired differences in nodule detection between MRI sequences.

**Results:**

Thirty-six patients were prospectively enrolled. One hundred forty-nine nodules (100 solid/49 subsolid) with mean size 10.8 mm (SD = 9.4) were included in the analysis. There was substantial interobserver agreement (k = 0.7, *p* = 0.05).

Detection for all nodules, solid and subsolid nodules was respectively; UTE: 71.8%/71.0%/73.5%; VIBE: 61.6%/65%/55.1%; HASTE 72.4%/72.2%/72.7%. Detection rate was higher for nodules > 4 mm in all groups: UTE 90.2%/93.4%/85.4%, VIBE 78.4%/88.5%/63.4%, HASTE 89.4%/93.8%/83.8%. Detection of lesions ≤4 mm was low for all sequences. UTE and HASTE performed significantly better than VIBE for detection of all nodules and subsolid nodules (diff = 18.4 and 17.6%, *p* = < 0.01 and *p* = 0.03, respectively). There was no significant difference between UTE and HASTE. There were no significant differences amongst MRI sequences for solid nodules.

**Conclusions:**

Lung MRI shows adequate performance for the detection of solid and subsolid pulmonary nodules larger than 4 mm and can serve as a promising radiation-free alternative to CT.

## Background

Lung cancer is the second most frequent cancer worldwide and is the leading cause of cancer-related mortality [1]. In the last decades, multiple early lung cancer detection initiatives based on screening at-risk populations with low-dose chest CT have been undertaken with the rationale that early stage diagnosis and subsequent treatment results in a more favorable prognosis. In 2011, the US national lung screening trial (NLST) showed a 20% lung cancer mortality reduction in a high risk population that underwent annual screening with low-dose chest CT as compared to chest x-ray [2]. In 2016, the pilot UK lung cancer screening (UKLS) demonstrated that screening an at-risk population allows for the detection of lung cancer in early stages and that this intervention is cost effective [3].

However, low dose chest CT inherently involves an augmented exposure to medical radiation which increases the long-term risk of radiation-induced cancer [4, 5]. It has been estimated that the lifetime attributable risk of low dose chest CT screening to develop lung cancer ranges from 5.5-8.8 per 10,000 individuals after 10 years, and for other major cancers to 1.4 to 2.6 per 10,000 individuals [6].

Pulmonary imaging with MRI was historically limited by low proton density, rapid signal decay and respiratory motion artifact. However, technical developments achieved by stronger gradients, increased field homogeneity, parallel imaging reconstruction and shorter echo times has made lung MRI an interesting alternative to CT [7, 8].

A free-breathing 3D radial ultrashort echo time (UTE) technique has shown promising results in the assessment of pulmonary disease and specifically in the detection of pulmonary nodules, given that shorter echo times reduce magnetic susceptibility artifacts in the soft tissue-air interfaces [9]. Similarly, single-shot fast-spin-echo sequences have also shown high sensitivity for the detection of pulmonary nodules greater than 5 mm [10]. Clinical and experimental studies have shown a threshold detection size of 3-4 mm for solid nodules and detection rates ranging from 60 to 90% for 5-8 mm lesions and nearly 100% for nodules over 8 mm [7, 11, 12].

The role of lung MRI imaging in lung cancer screening or as a surveillance tool still needs to be defined, and more evidence is needed at this early stage of investigation. Evidence for part solid and ground glass nodules (“adenocarcinoma spectrum lesions”) is particularly sparse. The main objective of the present work is to study the accuracy of T1 gradient-echo (Volumetric interpolated breath-hold examination or VIBE), ultrashort time echo (UTE) and single-shot T2 fast spin echo (Half fourier Single-shot Turbo spin-Echo or HASTE) sequences for the detection of pulmonary nodules. The secondary objective is to determine if there are differences in the detection rate of solid and subsolid nodules.

## Methods

### Patients

This study was approved by the Research Ethics Board of Sunnybrook Health Sciences Centre in Toronto, Canada, with informed consent obtained from all participants. Patients with Stage I non-small cell lung cancer (NSCLC) eligible for stereotactic body radiotherapy (SBRT) were prospectively enrolled between April 22, 2017 and June 1, 2021 as part of another study. Each patient had a baseline chest CT, which demonstrated the known primary malignancy and a variable number of additional small pulmonary nodules.

### Image acquisition

Patients underwent a pre-treatment lung MRI using a 3 T scanner (Magnetom Prisma, Siemens Healthcare) equipped with an 18-element body coil. The protocol included ultra-short time echo (UTE), spoiled 3D T1 gradient echo (VIBE), and single-shot turbo spin echo (HASTE) sequences. The image acquisition parameters for each sequence are summarized in Table 1. Different vendor names for each MRI sequence are highlighted in Table 2.

**Table 1:** Image acquisition parameters for each sequence in the study

	UTE	VIBE	HASTE
Orientation	Coronal	Transverse	Transverse
TR (ms)	2.57	3.57	600
TE (ms)	0.05	1.6	32
Flip angle (°)	5.5	9.0	160
FOV (mm)	600	380	400
Matrix (mm)	288 × 288	256 × 256	256 × 256
Slice thickness (mm)	2.5	3	5
Parallel imaging	none	CAIPIRINHA ×2	GRAPPA ×3
Partial Fourier	6/8	7/8	4/8
Gating	–	–	–
Breath-hold	inspiration	inspiration	inspiration
Acquisition time (min:s)	0:14 s	0:14 s	0:14 s

*TR* repetition time, *TE* Echo time, *FOV* Field of view

**Table 2:** MRI sequence name by vendor

Sequence	Siemens	GE	Phillips	Hitachi	Canon
Ultra-short echo time	UTE	3D ZTE	UTE	microTE	3D Ortho, Lung
Single-shot turbo spin echo	HASTE	SS-FSE	SSH-TSE	Single Shot FSE	FASE
Spoiled 3D GRE variants	VIBE	FAME/LAVA	THRIVE	TIGRE	3D QUICK

*UTE* Ultrashort echo time, *ZTE* Zero echo time, *HASTE* Half fourier Single-shot Turbo spin-Echo, *SS-FSE* single-shot fast spin-echo, *SSH-TSE* Single-shot Turbo Spin-echo, *FES* Fast spin-echo, *FASE* Fast advanced spin-echo, *VIBE* Volumetric Interpolated Breath-hold examination, *FAME* Fast Acquisition with Multi-phase Efgre3D, *LAVA* Liver Acquisition with Volume Acceleration, *THRIVE* T1 weighted High Resolution Isotropic Volume Examination, *TIGRE* T1-weighted Gradient Echo

For UTE and VIBE, the field of view (FOV) included the whole chest, whereas the HASTE FOV was limited to the lung parenchyma adjacent to the known primary malignancy given institutional magnet time restrictions. The HASTE and T1 GRE (VIBE) scans were acquired in the axial plane. Short rectangular pulses, a center-out acquisition and variable TE encoding were applied to achieve an effective TE of 50 μs. To reduce the number of through-plane phase-encoding steps in UTE, images were acquired in the coronal plane [13].

After the completion of lung MRI, all patients had surveillance chest CT as part of their standard-of-care, 3-4 months after SBRT.

### Image interpretation

All noncalcified pulmonary nodules were identified and measured on the baseline CT by two fellowship-trained thoracic radiologists in consensus (R1 and R2; 4 and 17 years of experience) and the maximum diameter was recorded. Nodules were categorized into groups according to their density on CT; solid or subsolid (including pure ground-glass and part-solid nodules); and size > 4 mm or ≤ 4 mm, and this evidence was used as the gold standard.

The baseline CTs were done in multiple different centers as patients were referred to our tertiary hospital for treatment. Hence, the CT technical parameters were not explicitly controlled and were performed with or without intravenous contrast and in different scanners (16 to 64-multidetector CTs) across the cohort of participants. Images were reconstructed using a soft tissue and a lung kernel, with a slice thickness ranging between 1.25 to 3 mm. Readers ensured that all baseline examinations were of diagnostic quality and encompassed the whole chest before being included in the study. The nodules were cross-referenced in the follow-up chest CT to ensure that they were present at the time of the lung MRI.

The included pulmonary nodules were classified as present or absent on the different MRI sequences by R1 and R2 independently. Radiologists had access to the baseline CT images while reading the MRI images, allowing for a side-by-side comparison.

### Statistical analysis

Detection of nodules for each different group (according to density and size) was expressed in percentages with 95% confidence intervals (CIs). Interobserver agreement was determined using the simple Kappa coefficient.

Continuous variables were reported as means ± standard deviation or as medians and range, as appropriate. Paired differences were compared using nonparametric Mann-Whitney U tests.

The McNemar test was used to evaluate paired differences in nodule detection between MRI imaging techniques. A *p*-value lower than 0.05 was considered significant. Choice of non-parametric test was to accommodate positively skewed variables. All statistical analyses were carried out using SAS 9.4 software (Cary, NC, USA).

## Results

### Patients

Patient demographics and characteristics are listed in Table 3. Thirty-six patients, 20 males and 16 females were prospectively enrolled. The mean age of the participants was 75.0 (SD ± 8.3) years. Mean time between the baseline CT and lung MRI was 43.2 ± 13.2 days. The majority of the primary lung cancers were solid (*n* = 28) with 8 cancers being subsolid. All baseline CTs were classified as providing diagnostic quality, with no major artifacts. The most frequent additional findings were emphysema (*n* = 19) and minor small airway changes such as tree-in-bud or mucus plugging (*n* = 9). Four patients had focal post-radiation fibrosis from a previously treated malignancy.

**Table 3:** Patient demographics and characteristics

Sex	**Male**	**Female**
	20 (55.6%)	16 (44.4%)
Mean age	75.8 ± 8.3 years	
Time between baseline CT and lung MRI	43.2 ± 13.2 days	
Mean number of nodules by patient	4.1 ± 2.6	
Additional findings in baseline CT	Emphysema = 19 Tree-in-bud opacities and mucus plugging = 9 Previous coronary artery bypass graft surgery = 5 Post-radiation fibrosis = 4	

### Pulmonary nodules

The nodule characteristics are listed in Table 4. A total of 149 nodules - 100 solid (67.1%) and 49 (32.9%) subsolid - identified on the baseline CT were included in the analysis. Given the limitations of HASTE, only 124 nodules could be potentially visualized on this sequence as some nodules were not included in the FOV. All the nodules were included in the FOV of UTE and VIBE.

**Table 4:** Nodule characteristics

Nodule characteristics			
All nodules (*n* = 149)			
Mean diameter	10.8 (SD ± 9.4) mm		
MEDIAN DIAMETER	7 mm (IQR = 10 mm)		
Location (lobes)	RUL = 52 (34.9%) RML = 11 (7.4%) RLL = 27 (18.1%) LUL = 39 (26.2%) LLL = 20 (13.4%)		
Nodules > 4 mm (n)	102		
Nodules ≤4 mm (n)	47		
Nodules by density			
	Solid (*n* = 100)	Subsolid (*n* = 49)	*P* value
Mean diameter	10.4 (SD ± 9.6) mm	11.5 (SD ± 8.8) mm	0.09
Median DIameter	6 mm (IQR 10 mm)	9 mm (IQR 10 mm)	–
Nodules > 4 mm (n)	61	41	–
Nodules ≤4 mm (n)	39	8	–
Nodules by sequence^a^			
	UTE and T1-GRE (n = 149)	T2-FSE (*n* = 124)	*P* value
Mean diameter	10.8 (SD ± 9.4) mm	11.5 (SD ± 9.9) mm	0.6
MEdian DIameter	7 mm (IQR 10 mm)	7 mm (IQR 9 mm)	–
Nodules > 4 mm (n)	102	86	–
Nodules ≤4 mm (n)	47	38	–

(^a^): Nodules included in the FOV of the corresponding sequences

The mean diameter of all nodules was 10.8 (SD ± 9.4) mm. There was no significant difference in size between solid and subsolid nodules [10.4 (SD ± 9.6) mm versus 11.5 (SD ± 8.8) mm respectively; *p* = 0.09]. There was no significant difference between nodules included in the limited FOV of HASTE and the nodules included in UTE and VIBE (VIBE/UTE = 10.8 (SD ± 9.4) mm; HASTE = 11.5 (SD ± 9.9) mm; *p* = 0.6) (Table 4).

### Nodule detection and sequence comparison

Table 5 lists the different detection rates in all sequences regarding the size and density of nodules, as well as detailed comparison among the different MRI sequences.

**Table 5:** Different detection proportions

	UTE	VIBE	HASTE	UTE vs VIBE *(P value)*	HASTE vs VIBE *(P value)*	UTE vs HASTE *(P value)*
All nodules	71.8% (107/149) *95% CI = 65-79%*	61.7% (92/149) *95% CI = 54-70%*	72.4% (89/123) *95% CI 64-80%*	< 0.01*	0.03*	0.79
*> 4 mm*	90.2% (92/102) *95% CI = 84-96%*	78.4% (80/102) *95% CI = 70-86%*	89.4% (76/85) *95% CI = 83-96%*	< 0.01*	< 0.01*	0.35
*≤ 4 mm*	31.9% (15/47) *95% CI 19-45%*	25.5% (12/47) *95% CI 13-38%*	34.2% (13/38) *95% CI = 19-49%*	0.19	0.32	0.48
Solid Nodules	71% (71/100) *95% CI = 62-80%*	65% (65/100) *95% CI = 56-74%*	72.2% (57/79) *95% CI 62-82%*	0.56	0.56	0.71
*> 4 mm*	93.4% (57/61) *95% CI 87-100%*	88.5% (54/61) *95% CI 80-97%*	93.8% (45/48) *95% CI 87-100%*	0.50	0.32	0.32
*≤ 4 mm*	35.9% (14/39) *95% CI 21-51%*	28.2% (11/39) *95% CI 14-42%*	38.7% (12/31) *95% CI 22-56%*	0.11	0.16	0.41
Subsolid Nodules	73.5% (36/49) *95% CI = 61-86%*	55.1% (27/49) *95% CI = 41-69%*	72.7% (32/44) *95% CI = 60-86%*	< 0.01*	0.03*	0.48
*> 4 mm*	85.4% (35/41) *95% CI 75-96%*	63.4% (26/41) *95% CI 49-78%*	83.8% (31/37) *95% CI 72-96%*	< 0.01*	< 0.01*	0.59
*≤ 4 mm*	12.5% (1/8) *95% CI 0-35%*	12.5% (1/8) *95% CI 0-35%*	14.2% (1/7) *95% CI 0-40%*	0.32	0.32	1.0

(*): significant; P value < 0.05

There was substantial interobserver agreement between the 2 readers (Kappa coefficient = 0.7; 95% CI = 0.62-0.77). Overall nodule detection (solid and subsolid nodules) for UTE was 71.8% (107/149), VIBE 61.7% (92/149) and HASTE 72.4% (89/123). The known primary lung malignancy was depicted by all the different MRI sequences in each case. Nodule detection markedly increased when nodules smaller than 5 mm were excluded from the analysis: UTE = 90.2% (92/102), VIBE = 78.4% (80/102), and HASTE = 89.4% (76/85). Detection was lower for smaller nodules (≤ 4 mm): UTE 31.9% (15/47), VIBE 25.5% (12/47), and HASTE 34.2% (13/38). Detection rate was significantly higher for UTE and HASTE when compared to VIBE for nodules of all size and for nodules larger than 4 mm (UTE vs VIBE: *p* = < 0.01; HASTE vs VIBE: *p* = 0.03 for all nodules and < 0.01 for nodules > 4 mm). There were no significant differences for smaller nodules (≤ 4 mm).

**Fig. 1:**
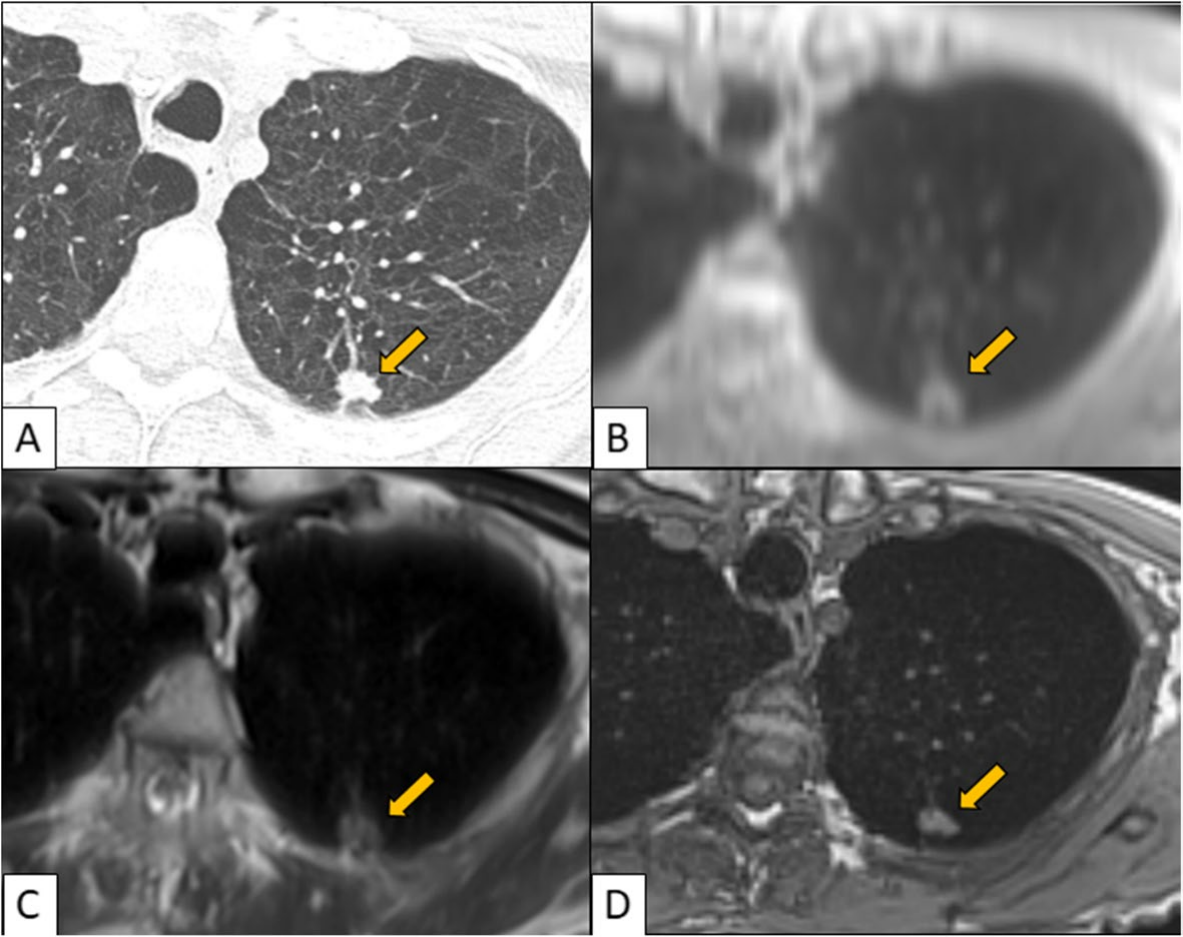
CT (**A**), UTE (**B**), HASTE (**C**), VIBE (**D**). Left upper lobe spiculated solid nodule is detected in a similar way in the 3 MR sequences: UTE, HASTE and VIBE

**Fig. 2:**
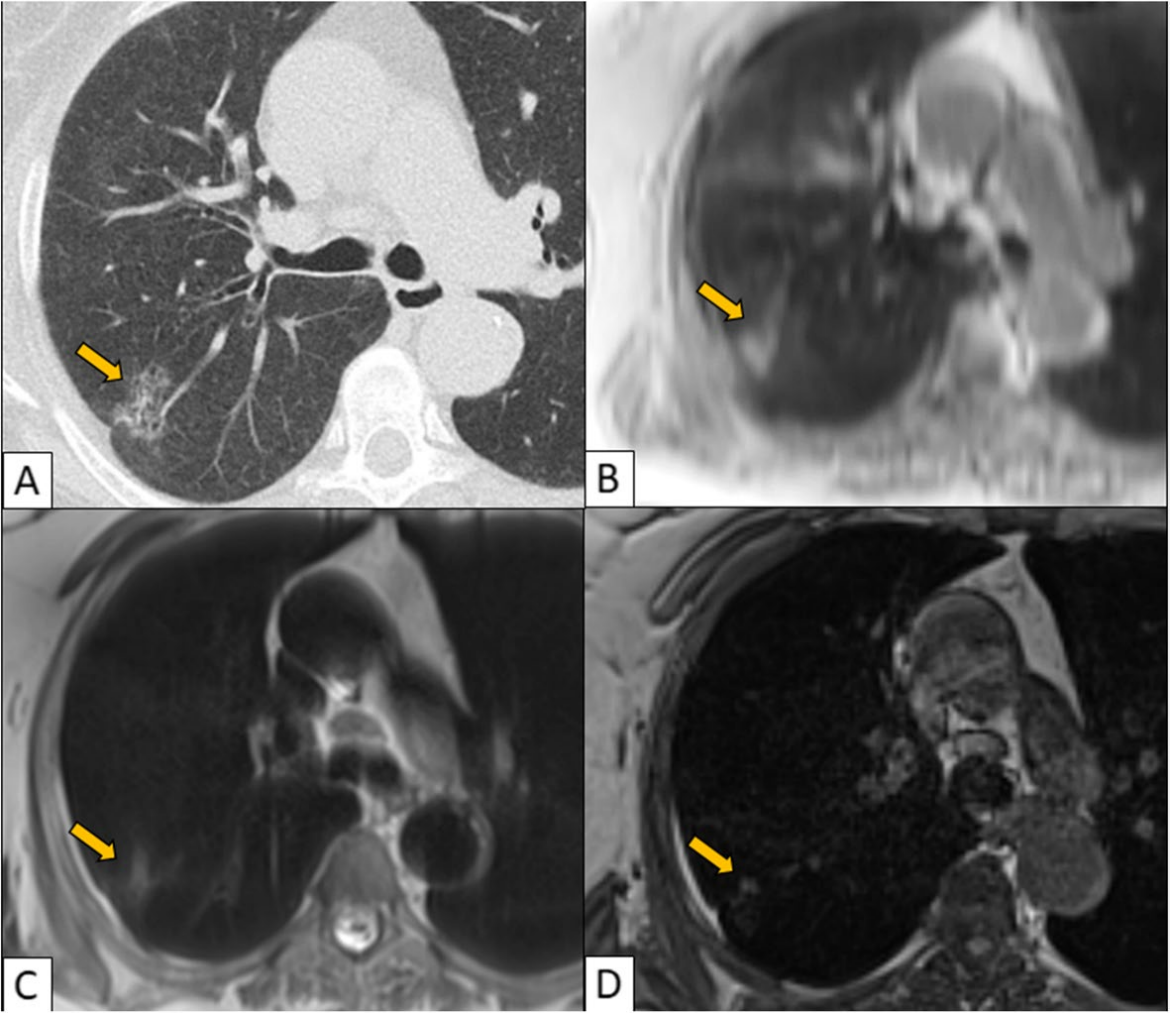
CT (**A**), UTE (**B**), HASTE (**C**), VIBE (**D**). Right upper lobe ground glass nodule is clearly detected in UTE and less well seen in HASTE. It is inconspicuous in VIBE

Detection of solid nodules was similar among all sequences and ranged from 65.0 - 72.2%. For solid nodules > 4 mm, detection rate was > 85% in all sequences. Detection of smaller solid nodules (≤ 4 mm) ranged from 28.2 - 38.7%. There were no significant differences between the three imaging sequences regarding the solid nodules (Fig. 1). UTE and HASTE performed equally well in the detection of subsolid nodules with rates comparable to the detection of solid nodules (73.5 and 72.7%, respectively), whereas VIBE showed a significantly lower detection rate (55.1%) (Fig. 2). For subsolid nodules > 4 mm, UTE and HASTE showed a detection rate > 80%, whereas VIBE had a significantly lower detection rate of 63.4%. UTE and HASTE performed significantly better than VIBE for subsolid nodules of all sizes and those > 4 mm (UTE vs VIBE: *p* = < 0.01; HASTE vs VIBE: *p* = 0.03 for all nodules and < 0.01 for nodules > 4 mm). Detection was low for nodules ≤4 mm in all 3 sequences, ranging from 12.5 - 14.2% and there were no significant differences amongst the 3 sequences. Only one nodule was detected in each sequence in this category.

## Discussion

This prospective study demonstrates, that lung MRI has the ability to detect small subsolid and solid nodules larger than 4 mm accurately and in a comparable way to thin section chest CT. Specifically, UTE and HASTE outperformed T1-GRE (VIBE) in all nodules irrespective of size and density, in all subsolid nodules irrespective of size, and in all nodules and subsolid nodules larger than 4 mm. In addition, there was substantial interobserver agreement for pulmonary nodule detection amongst the different MRI sequences.

Although chest CT remains the gold standard imaging modality for the detection of pulmonary nodules, a major caveat is radiation exposure, which is not negligible despite the broad application of low dose techniques and iterative reconstruction [14–16]. With the massification of chest CT, small sub-centimeter pulmonary nodules have become a frequent finding. These most often represent an incidental finding and are not clinically significant. There is a non-linear relationship between nodule size and probability of cancer [17]: the probability of cancer of a nodule smaller than 5 mm is approximately 0.4%, whereas it reaches 15.2% for nodules larger than 10 mm [18, 19]. In 2017, the Fleischner society updated their guidelines for the management of incidental pulmonary nodules [20] increasing the threshold of size requiring follow-up to 6 mm, compared to their first version published in 2005 [18] to reduce the number of unnecessary follow-up examinations. Still, many patients will require at least one follow-up CT to confidently exclude malignancy [20].

Significant technical advancements in the last 2 decades have established lung MRI as a complementary and promising radiation-free imaging modality for the detection of pulmonary nodules and thoracic oncology that has the potential to become an attractive alternative to CT.

The overall nodule detection rate of lung MRI in our study ranged between 72% (UTE and HASTE) and 62% (VIBE). Reported rates regarding HASTE are variable in previous studies, with Shroeder et al. reporting an overall nodule detection rate of 85.4% [21], whereas Cieswanowski et al. reported an overall nodule detection rate of 26% [22]. The authors attributed these discrepancies to different hardware and software settings [22]. Many researchers have used 3D T1-weighted GRE (VIBE) with Chandarana et al. reporting an overall nodule detection rate of 62% [23] and Dews et al. reporting a sensitivity of 88% [24]. In a previous study where the authors compared the performance of UTE versus standard and low dose chest CT, they reported a detection rate of 93% for all nodules [25].

The detection rate for all nodules larger than 4 mm in the present study was 89.4% with HASTE, 78.4% with VIBE and 90.2% with UTE. Shroeder et al. reported a detection rate of 86.3% with HASTE for nodules between 3 and 5 mm and 95.7% for nodules between 6 and 10 mm [21]. Cieszanowski et al. reported a rate of 20.3% with HASTE for nodules between 4 and 8 mm and 50% for nodules larger than 8 mm [22]. Dews et al. reported a detection rate of 87.2% with VIBE for nodules between 5 and 10 mm [24]. Olthof et al. reported a low rate of 25% for breath-hold VIBE for nodules between 5 and 10 mm [13], and Yu et al. reported a rate of 62% for breath-hold VIBE for nodules between 4 and 6 mm and 93% for free breathing radial VIBE [26]. Regarding nodules larger than 4 mm, Ohno et al. reported a rate of 74.1% for nodules 4-6 mm - compared to 71.8% in our study – and 94.7% for all solid nodules 6-8 mm – compared to 93.4% in the present study [25]. Ohno et al. did not assess nodules smaller than 4 mm [25].

The detection rate for subsolid nodules irrespective of size was 73.5% for UTE, 72.7% for HASTE and 55.1% for VIBE in the present study. When assessing subsolid nodules larger than 4 mm the detection rates increased to 85.4% for UTE, 83.8% for HASTE and 63.4% for VIBE. Interestingly although UTE performed slightly better than HASTE in the detection of all subsolid nodules and subsolid nodules larger than 4 mm, this did not reach statistical significance. Regarding subsolid nodules larger than 4 mm, Ohno et al. reported a rate of 33.3% with UTE for nodules between 4 and 6 mm and 100% for nodules between 6 and 8 mm [25]. In a study with a lung screening population, 7 out of 8 subsolid nodules were visible on T2-STIR and T2 but there is no information regarding their size [27]. Of note, none of the subsolid nodules in the same study were detected by THRIVE (3D-T1), which is equivalent to VIBE [27].

MRI Sequences with ultra-short TE have been introduced to mitigate the drawbacks of the shorter T2* relaxation times of the lung parenchyma compared to other tissues, secondary to inhomogeneous magnetic susceptibility of lung parenchyma resulting from its air/soft tissue interfaces [28]. Previous studies have shown that the UTE sequence can be used safely to detect significant detail of the lung parenchyma and interstitium comparable to CT including emphysema, interstitial lung disease and airway disease [29–31]. The present study showed comparable detection rate of subsolid nodules > 4 mm (85%) when compared to the only other study in the literature assessing subsolid nodule detection, where 81.8% rate was reported for subsolid nodules 6-8 mm [25]. The authors of that study suggested that free-radiation lung MRI imaging with UTE could play a significant role in detection and evaluation of nodule type in lung cancer screening, lung metastasis follow up as well as in the assessment of treatment response.

Studies have shown improved detection rates when using free breathing VIBE resulting in 94% detection rate compared to breath-hold VIBE (64.3%), however the scanning time is significantly increased from 14 sec to 7 min which might not be clinically applicable [13, 26]. In the present study, breath-hold VIBE had the lowest detection rate compared to UTE and HASTE.

HASTE is a single-shot turbo spin echo T2 weighted sequence and has been studied extensively for the detection of pulmonary nodules with comparable results to the present study [21, 32, 33], especially for nodules larger than 5 mm, however it has not been studied for detection of subsolid pulmonary nodules. In a study by Meier-Shroers et al., T2 weighted imaging with an acquisition time of 3.18 min. Was most reliable for the detection of pulmonary nodules ≥6 mm, however they did not evaluate HASTE [27].

There were limitations to this study. First, the number of patients was small, although the total number of nodules was relatively large when compared to other series [10, 11, 27], and smaller nodules were not excluded. Second, radiologists had access to the CT images while reading the MRI sequences, which may have caused bias towards overcalling pulmonary nodules on the MRI images. This could be assessed in future studies by blinding the readers to the CT images, simulating real-life clinical conditions. Third, there was a time interval between the baseline CT and lung MRI (43.2 ± 13.2 days). It is possible that some lesions might have slightly increased in size in the interval, although this is likely non-significant as the average doubling time (growth-rate) of malignant lesions is longer than this period, and benign lesions tend to remain stable [34, 35]. Fourth, as HASTE did not include the whole chest, some nodules were excluded from the analysis for this sequence in particular. Fifth, we did not assess morphological characteristics of the nodules such as shape, border, location or calcification. Lastly, a number of nodules did not have histologic confirmation and therefore we did not attempt to differentiate malignant from benign nodules.

## Conclusion

In conclusion, this study has shown that clinically significant solid and subsolid nodules larger than 4 mm can be accurately detected by lung MRI using breath-hold UTE and HASTE that could be completed in less than 5 min of magnet time [12]. Fast non-contrast lung MRI is gaining its role in the routine clinical practice as a radiation-free alternative to CT for nodule detection and could be used in lung cancer screening, follow-up of pulmonary nodules, and in the metastatic work-up of oncologic patients.

## Abbreviations

GRE: Gradient-echo
VIBE: Volumetric interpolated breath-hold examination
UTE: Ultrashort time echo
MRI: Magnetic Resonance
HASTE: Half fourier Single-shot Turbo spin-Echo
CT: Computed Tomography
NLST: National Lung Screening Trial
UKLS: United Kingdom lung cancer screening
NSCLC: Non-small cell lung cancer
SBRT: Stereotactic body radiotherapy
FOV: Field of view
